# Research advances and clinical management of drug interactions between voriconazole and antituberculosis agents

**DOI:** 10.3389/fphar.2026.1962108

**Published:** 2026-10-21

**Authors:** Yijie Fu, Zhiqiang Lin

**Affiliations:** 1 Department of Pharmacy, Quanzhou First Hospital Affiliated to Fujian Medical University, Quanzhou, Fujian, China; 2 College of Pharmacy, Fujian Medical University, Fuzhou, Fujian, China

**Keywords:** antituberculosis drugs, drug interactions, pharmacokinetics, therapeutic drug monitoring, voriconazole

## Abstract

**Background:**

When tuberculosis is complicated by fungal infection, voriconazole and antituberculosis drugs may need to be used in combination, and drug interactions can affect efficacy and safety.

**Methods:**

This structured narrative review used a systematic literature search of PubMed, Embase, Web of Science, and the China National Knowledge Infrastructure (CNKI) from database inception to May 2026, together with predefined eligibility criteria and evidence stratification. Clinical pharmacokinetic, therapeutic drug monitoring (TDM), observational, case-report, and case-series studies were eligible as the primary evidence base. Relevant *in vitro* studies were reviewed only as mechanistic, hypothesis-generating evidence and were not treated as primary clinical evidence. Because of substantial heterogeneity in study design, populations, interventions, pharmacokinetic endpoints, and reporting completeness, the findings were synthesized narratively rather than quantitatively. The direction and potential clinical significance of interactions and key considerations for TDM were summarized by evidence type.

**Results:**

A total of 2,198 records were identified, and 17 primary clinical studies were included. Most were case reports or small retrospective studies, with limited paired antituberculosis-drug exposure data and clinical outcomes; generalizability is therefore limited. Existing clinical pharmacokinetic evidence is concentrated on rifamycins. Rifampicin markedly reduces voriconazole exposure and its induction may persist after discontinuation, so coadministration should be avoided. Rifapentine can also substantially lower voriconazole concentrations, with interindividual variability in the offset of induction; dose adjustment and re-initiation after withdrawal require serial TDM. Rifabutin has a bidirectional interaction: it can reduce voriconazole exposure, while voriconazole may increase exposure to rifabutin and its active metabolite. Current U.S. voriconazole labeling lists rifabutin coadministration as contraindicated, and rare off-label case experience does not establish it as a safe alternative. For isoniazid, ethambutol, linezolid, and other non-rifamycin agents, available evidence is mechanistic or hypothesis-generating and does not establish causal interactions or justify empirical voriconazole dose adjustment.

**Conclusion:**

Management should prioritize avoidance of voriconazole coadministration with rifampicin and rifabutin in accordance with current labeling, and avoidance of rifapentine where feasible. If rifapentine exposure cannot be avoided or has recently stopped, serial TDM should guide voriconazole dosing. For non-rifamycin antituberculosis drugs, monitoring should be matched to the suspected risk (exposure variability, QT prolongation, hepatotoxicity, or optic, neurologic, or hematologic toxicity) rather than based on unvalidated empirical dose changes.

## 1 Introduction

Voriconazole is a second-generation triazole antifungal agent that has become one of the first-line treatments for invasive aspergillosis because of its activity against *Aspergillus* and other pathogenic fungi (Herbrecht et al., 2002). Tuberculosis remains a major infectious disease worldwide (Saukkonen et al., 2025), and standard treatment commonly involves isoniazid, rifampicin, ethambutol, and pyrazinamide. Against the background of chronic pulmonary structural destruction, impaired immune function, or long-term anti-infective therapy, patients with tuberculosis may develop fungal coinfection such as aspergillosis. A systematic review (Hosseini et al., 2020) showed that the prevalence of *Aspergillus* coinfection among patients with pulmonary tuberculosis in Asia and Africa was approximately 15.4%. Therefore, when invasive or chronic pulmonary aspergillosis is diagnosed or highly suspected, some patients need to use voriconazole during antituberculosis treatment.

However, the combined use of these two classes of drugs is often limited by pharmacokinetic interactions (Yew, 2002). Voriconazole is mainly metabolized by the cytochrome P450 (CYP) enzymes CYP2C19, CYP2C9, and CYP3A4, and its plasma concentrations vary widely between individuals (Hyland et al., 2003), with a relatively narrow therapeutic window. Among antituberculosis drugs, rifampicin and rifapentine can markedly reduce voriconazole exposure, whereas rifabutin carries a bidirectional interaction risk: it reduces voriconazole exposure while voriconazole can increase exposure to rifabutin itself. Existing evidence is mainly derived from case reports, retrospective studies, small-sample pharmacokinetic studies, and limited *in vitro* data, and its strength and generalizability are heterogeneous (Li et al., 2017). This review summarizes the pharmacokinetic interactions between voriconazole and commonly used antituberculosis drugs, with emphasis on the strength of evidence, clinical risk, and therapeutic drug monitoring (TDM)-based management strategies for different drug combinations, to provide a reference for individualized pharmacotherapy in patients with these coexisting conditions. In particular, we distinguish interactions supported by human pharmacokinetic data from potential risks inferred mainly from mechanistic considerations, *in vitro* studies, or safety signals. Accordingly, this article is presented as a structured narrative review based on a systematic literature search, rather than as a formal systematic review or meta-analysis.

## 2 Methods and quality appraisal

### 2.1 Data sources and search strategy

This study was designed as a structured narrative review using a systematic literature search, predefined eligibility criteria, narrative synthesis, and evidence stratification. It was not conducted as a formal systematic review or meta-analysis. PubMed, Embase, Web of Science, and the China National Knowledge Infrastructure (CNKI) were searched from database inception to May 2026 to identify clinical and mechanistic evidence on interactions between voriconazole and antituberculosis drugs. The search strategy was designed with reference to the commonly used and second-line antituberculosis drug lists in the World Health Organization comprehensive guidelines for tuberculosis. “Voriconazole” was paired with “antitubercular agents,” “isoniazid,” “rifampicin/rifampin,” “pyrazinamide,” “ethambutol,” “rifapentine,” “rifabutin,” “bedaquiline,” “pretomanid,” “linezolid,” “moxifloxacin,” “levofloxacin,” “delamanid,” “clofazimine,” “cycloserine,” “terizidone,” “ethionamide,” “protionamide,” “para-aminosalicylic acid,” “amikacin,” “streptomycin,” “imipenem-cilastatin,” “meropenem,” and other keywords in pairwise combinations. Disease-related terms such as “tuberculosis” and “invasive aspergillosis” were also combined to improve coverage of tuberculosis complicated by invasive fungal infection. Corresponding Chinese drug names, disease names, and terms such as “drug interaction,” “pharmacokinetics,” and “therapeutic drug monitoring” were used for the CNKI search.

### 2.2 Inclusion and exclusion criteria

To reduce the risk of missing relevant reports, the reference lists of included studies, relevant guidelines, and drug prescribing information were also manually screened. After duplicate removal, title/abstract screening, full-text eligibility assessment, and data extraction were independently performed by YF and ZL using a predefined standardized data-extraction form. Disagreements were resolved through discussion and consensus between the two reviewers. The inclusion criteria were: (i) studies involving the combined use of voriconazole with one or more antituberculosis drugs; (ii) studies reporting plasma concentrations, pharmacokinetic parameters, TDM results, dose-adjustment processes, or clinical outcomes related to the interaction; and (iii) pharmacokinetic studies in healthy volunteers, patient TDM studies, case reports, case series, retrospective cohort studies, and prospective observational studies. *In vitro* experiments, drug prescribing information, and guidelines were used mainly to explain possible mechanisms or supplement clinical management evidence and were not considered primary evidence for clinical conclusions.

The exclusion criteria were duplicate publications; studies from which valid pharmacokinetic or TDM information could not be extracted; studies that only mentioned coadministration without analyzing the interaction; studies whose population, intervention, or outcome was not consistent with the topic of this review; reviews, editorials, and guidelines that did not provide original case or pharmacokinetic data; animal experiments; and purely *in vitro* studies that did not provide clear mechanistic support for the clinical discussion.

### 2.3 Data extraction and quality assessment

The following information was extracted from the included studies: first author, year of publication, study type, study population, sample size, age, sex, body weight or body mass index (BMI), type of antituberculosis drug, route and dose of voriconazole administration, timing of combination or withdrawal, voriconazole plasma concentration or pharmacokinetic parameters, dose adjustment process, clinical efficacy, and adverse reactions. For studies with incomplete reporting, information was extracted as available in the original text, and unreported items were marked.

Because the included studies were heterogeneous in study design, underlying disease, concomitant medication, dosing regimen, sampling time, and pharmacokinetic endpoints, no meta-analysis was performed. The reports did not provide a common effect measure or sufficiently comparable time points for quantitative pooling, and most were case reports, case series, or retrospective observational studies. We therefore used a descriptive narrative synthesis with evidence stratification. Case reports were appraised with the Joanna Briggs Institute (JBI) case-report checklist, case series or single-arm observational studies with the JBI case-series checklist, and retrospective cohort studies with the Newcastle–Ottawa Scale (NOS). JBI scores describe reporting completeness only; they do not measure evidence certainty, causal strength, generalizability, or recommendation strength.

NOS scores were categorized as 7–9 stars (high methodological quality), 5–6 stars (moderate), and 0–4 stars (low). For the JBI checklists, prespecified descriptive thresholds based on the proportion of “Yes” responses were used to describe reporting completeness: ≥70% indicated relatively complete reporting, 50%–69% moderate reporting completeness, and <50% limited reporting completeness. JBI scores were calculated as the number of “Yes” responses divided by the number of applicable checklist items; “Not applicable” (NA) responses were excluded from the denominator, whereas “No” and “Unclear” responses remained applicable items. The case-report checklist contains eight items (Q1–Q8), and the case-series checklist contains ten items (Q1–Q10). These thresholds and scores were used for descriptive presentation and should not be interpreted as JBI-endorsed ratings of evidence certainty. JBI checklist results indicate reporting quality and completeness, not certainty of evidence, causal strength, generalizability, or the strength of a clinical recommendation. Accordingly, even well-reported case reports and case series were considered low-level evidence for causal inference because they lack control groups and remain susceptible to confounding, selective reporting, and publication bias.

## 3 Results

### 3.1 Literature screening results

Database searches yielded 2,198 records: 371 from PubMed, 1,101 from Embase, 679 from Web of Science, and 47 from CNKI. After removal of 615 duplicates, 1,583 records entered title and abstract screening; 1,451 were excluded at this stage. Full texts were assessed for 132 records, of which 115 were excluded after eligibility assessment, leaving 17 studies for the narrative synthesis. The full-text exclusions were: insufficient detail to extract data (n = 12), review (n = 12), irrelevant drug-interaction focus (n = 46), animal experimentation (n = 2), not the study drug (n = 36), unable to locate the full text (n = 5), and wrong comparator (n = 2); these categories sum to 115. The included evidence was interpreted according to an explicit hierarchy. Direct pharmacokinetic studies were considered the most informative for quantifying changes in voriconazole exposure. Observational and therapeutic drug monitoring (TDM) studies provided clinically relevant but less controlled evidence. Case reports and case series were useful for identifying temporal associations, unusual toxicity, and possible recovery patterns, but had limited causal inferential strength. Pharmacovigilance analyses were treated as signal-detection evidence and could not establish causality or define the direction of pharmacokinetic change. Mechanistic and *in vitro* studies were considered hypothesis-generating. Guidelines and product labeling were used as regulatory or clinical-management context rather than as original evidence of the interaction. The literature screening flow is shown in Figure 1.

**FIGURE 1:**
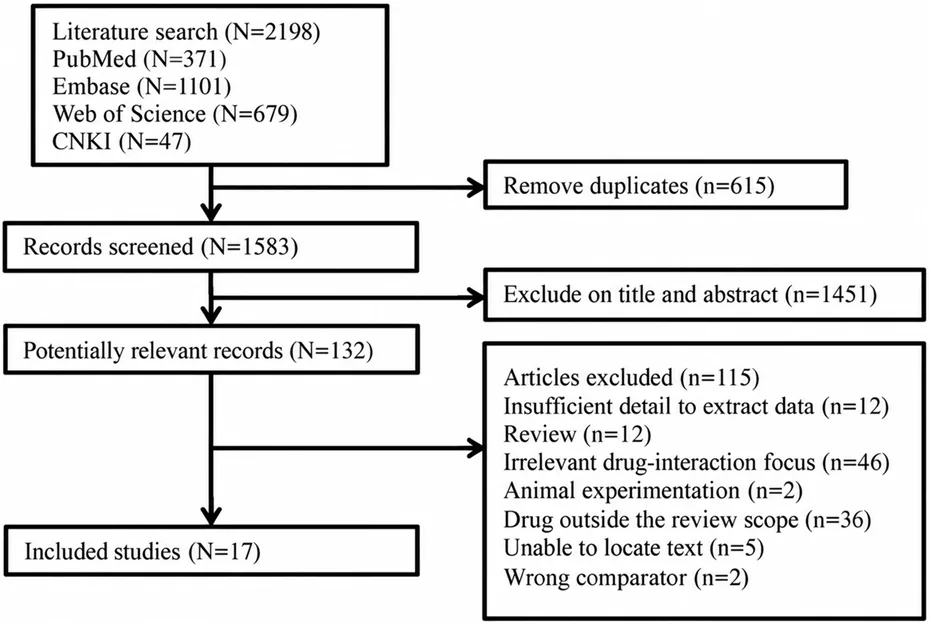
Flow chart depicting the selection process of studies included in the structured narrative review.

### 3.2 Basic characteristics of the included studies

For interpretation of concentrations, 1.0–5.5 mg/L was used as a commonly cited serum or plasma voriconazole trough reference range in the included reports (Pascual et al., 2008; Dolton et al., 2012; Park et al., 2012; Ashbee et al., 2014). This is not a universal therapeutic threshold: targets vary with infection site, organism, assay, treatment phase, toxicity risk, and guideline or institutional practice (Ashbee et al., 2014). Central nervous system infection may require a different exposure target, and trough concentration should not be conflated with maximum concentration (Cmax), area under the concentration-time curve (AUC), or apparent oral clearance (CL/F). Most included studies measured voriconazole trough concentrations; a minority also reported Cmax or AUC (Geist et al., 2007).

Overall, existing clinical evidence is highly concentrated on the interaction between rifamycin drugs and voriconazole. Rifampicin and rifapentine have been examined in more studies, and most of these studies provide TDM data during combination or after withdrawal; rifabutin has been examined in fewer studies, but its bidirectional interaction and safety risk are clinically important. By contrast, there is little direct, extractable clinical pharmacokinetic evidence for interactions between voriconazole and other first-line or second-line antituberculosis drugs.

### 3.3 Methodological and reporting quality appraisal

Among the cohort and retrospective observational studies included in the main analysis, NOS methodological quality was mainly moderate to high: three studies were rated high (Na et al., 2021; Chen et al., 2023; Lu et al., 2024), and three were rated moderate (Cojutti et al., 2016; Lu et al., 2019; Kim et al., 2025). Using the prespecified descriptive JBI thresholds, all 11 case reports and case series met the category for relatively complete reporting. This designation refers only to the completeness and clarity of reporting; it does not indicate high-certainty evidence, generalizability, or confirmed causality. Because case reports and case series lack control groups and are vulnerable to confounding, selective reporting, and publication bias, their inferential strength remained limited regardless of reporting completeness. Some case series also had uncertainty regarding consecutive inclusion, complete inclusion, or statistical analysis. The appraisal results are shown in Tables 1, 2.

**TABLE 1:** NOS quality assessment of cohort studies included in the main analysis.

Study	Design/Sample	S1	S2	S3	S4	C	O1	O2	O3	Score	Quality
Cojutti PG et al., 2016	Retrospective TDM cohort; rifampicin-related cases n = 2	1	0	1	1	1/2	1	1	0	6/9	Moderate
Lu H et al., 2019	Single-arm/retrospective study; rifampicin coadministration and post-withdrawal monitoring	1	0	1	1	0/2	1	1	1	6/9	Moderate
Chen TT et al., 2023	Retrospective cohort; rifampicin group n = 15, non-rifampicin group n = 17	1	1	1	1	1/2	1	1	1	8/9	High
Na et al., 2021	Cohort study; rifampicin n = 4, rifapentine n = 14, non-rifamycin comparison group	1	1	1	1	0/2	1	1	1	7/9	High
Lu H et al., 2024	Retrospective cohort; rifampicin/rifapentine coadministration and concentration recovery after withdrawal	1	1	1	1	1/2	1	1	1	8/9	High
Kim Y et al., 2025	Retrospective cohort; voriconazole plus rifabutin n = 6	1	0	1	1	0/2	1	1	1	6/9	Moderate

NOS, abbreviations: S1, representativeness of the exposed cohort; S2, selection of the non-exposed cohort; S3, ascertainment of exposure; S4, outcome not present at study start; C, comparability (maximum 2 stars); O1, outcome assessment; O2, adequate follow-up duration; O3, adequacy of follow-up.

**TABLE 2:** JBI appraisal of reporting completeness, not evidence certainty, for case reports and case series included in the main analysis.

Study	Drug(s)	Study type	JBI checklist	Q1	Q2	Q3	Q4	Q5	Q6	Q7	Q8	Q9	Q10	Score	Reporting completeness
Geist MJ et al., 2007	Rifampicin	Case report	Case report	U	Y	Y	Y	Y	Y	Y	Y	NA	NA	7/8	Relatively complete
Farrokh S & Avdic E, 2019	Rifampicin	Case report	Case report	Y	Y	Y	Y	Y	Y	Y	Y	NA	NA	8/8	Relatively complete
Wang ML et al., 2019	Rifampicin	Case report	Case report	Y	Y	Y	Y	Y	Y	Y	Y	NA	NA	8/8	Relatively complete
Lin ZQ et al., 2020	Rifampicin	Case report	Case report	Y	Y	Y	Y	Y	Y	Y	Y	NA	NA	8/8	Relatively complete
Lin ZQ et al., 2021	Rifampicin	Case series	Case series	Y	Y	Y	U	U	Y	Y	Y	Y	NA	7/9	Relatively complete
Sternberg CA et al., 2025	Rifampicin/Rifabutin	Case report	Case report	Y	Y	Y	Y	Y	Y	Y	Y	NA	NA	8/8	Relatively complete
Ye LB et al., 2020	Rifapentine	Case report	Case report	Y	Y	Y	Y	Y	Y	Y	Y	NA	NA	8/8	Relatively complete
Ling GM et al., 2023	Rifapentine	Case report	Case report	Y	Y	Y	Y	Y	Y	Y	Y	NA	NA	8/8	Relatively complete
Chen T et al., 2026	Rifapentine	Case report	Case report	Y	Y	Y	Y	Y	Y	Y	Y	NA	NA	8/8	Relatively complete
Schwiesow JN et al., 2008	Rifabutin	Case report	Case report	Y	Y	Y	Y	Y	Y	Y	Y	NA	NA	8/8	Relatively complete
Orssaud C et al., 2021	Ethambutol	Case report	Case report	Y	Y	Y	Y	Y	Y	Y	Y	NA	NA	8/8	Relatively complete

For JBI case reports, Q1–Q8 indicate: demographic characteristics, patient history/timeline, current clinical condition, diagnostic assessment, intervention/treatment procedure, post-intervention clinical condition, adverse or unanticipated events, and takeaway lessons. For the JBI case-series checklist, Q1–Q10 indicate: clear inclusion criteria, valid measurement of condition, valid case identification, consecutive inclusion, complete inclusion, demographics, clinical information, outcomes/follow-up, reporting of site/clinic information, and appropriate statistical analysis. Y = Yes; N = No; U = Unclear; NA = Not applicable. Scores are presented as Yes responses/applicable items; NA responses were excluded from the denominator. Thus, the case-report checklist has an eight-item denominator, the case-series checklist has a ten-item denominator when all items apply, and (Lin et al., 2021) is scored as 7/9 because Q10 was not applicable. JBI findings describe reporting completeness and should not be interpreted as evidence-certainty or causal-strength ratings.

### 3.4 Effects of rifamycin antituberculosis drugs on voriconazole pharmacokinetics

Clinical evidence with extractable voriconazole plasma concentrations or pharmacokinetic parameters is mainly concentrated on rifamycin drugs. In U.S. Food and Drug Administration (FDA) drug–drug interaction guidance, a strong inducer is generally defined by a ≥80% decrease in the AUC of a sensitive index substrate; this definition is not a formal classification of rifampicin based specifically on voriconazole. The approximately 96% reduction in voriconazole AUC reported with rifampicin is nevertheless consistent with a clinically strong induction effect (Food and Drug Administration, 2024; VFEND, 2026). Rifabutin and rifapentine also reduce voriconazole exposure, but differences in magnitude, duration, and bidirectional risk require serial TDM rather than assuming identical inducer strength.

#### 3.4.1 Rifampicin

The interaction between rifampicin and voriconazole is the best characterized and manifests as a marked reduction in voriconazole exposure. The case report (Geist et al., 2007) described a patient with acute myeloid leukemia in whom the first single oral dose of voriconazole 400 mg produced a C_max_ of 3.92 mg/L and an AUC_0-inf_ of 27.4 h·mg/L; after 36 days of voriconazole therapy with 30 days of concomitant rifampicin, total voriconazole exposure decreased by approximately 99%, with C_max_ falling to 0.038 mg/L and AUC_0–12 h_ only 0.145 h·mg/L. At the same time, the metabolite-to-parent ratios of the N-oxide, hydroxy-voriconazole, and dihydroxy-voriconazole increased approximately 45-fold, 178-fold, and 422-fold, respectively, supporting induction of voriconazole metabolism by rifampicin.

Retrospective TDM studies further confirmed the clinical significance of this interaction. A retrospective TDM study (Lu et al., 2019) included 18 patients receiving voriconazole and rifampicin; the voriconazole regimen was mainly 0.2 g every 12 h (15 patients), with two patients receiving a 0.3 g every 12 h loading dose on day 1 followed by 0.2 g every 12 h, and one patient receiving 0.3 g every 12 h. The voriconazole concentration was below 1.0 mg/L in 94.44% of patients, and 72.22% of concentrations were below the lower limit of quantification (0.16 mg/L). A second retrospective study (Chen et al., 2023) included 32 patients who received voriconazole and rifampicin concurrently or sequentially and underwent TDM: 15 in the concurrent combination group and 17 in the non-concurrent group. The loading doses were (5.87 ± 1.25) mg/kg and (5.41 ± 1.10) mg/kg, and the maintenance doses were (4.00 ± 0.58) mg/kg and (4.03 ± 0.32) mg/kg, respectively. In the combination group, the mean trough concentration was (1.58 ± 3.01) mg/L, 66.7% of concentrations were below the lower limit of the target range, and the target attainment rate was 26.7%; in the non-concurrent group, the mean trough concentration was (3.67 ± 2.44) mg/L, no patient was below the lower limit, and the attainment rate was 82.4%. These results indicate that even with conventional loading and maintenance doses, rifampicin markedly reduces voriconazole trough concentrations, and conventional dose escalation cannot reliably offset its induction effect.

The inducing effect of rifampicin persists for several days or more after withdrawal, which is easily underestimated when voriconazole is restarted. In one cohort (Lu et al., 2019), 91.67% of specimens collected within 6 days after withdrawal were below 1.0 mg/L, whereas 83.33% of specimens collected at 7 days or more were above 1.0 mg/L. In a later cohort (Lu et al., 2024), mean Cmin increased from (0.36 ± 0.34) mg/L at 1–3 days and (0.49 ± 0.56) mg/L at 4–6 days to (1.34 ± 0.72) mg/L at 7 days or more. Individual cases nevertheless suggested persistence for 10–13 days or longer. These data support approximately 7 days as an observed minimum recovery interval in some cohorts, not a biological guarantee; residual induction and the effects of illness, inflammation, liver function, genotype, adherence, and concomitant drugs may extend or shorten recovery.

Therefore, the rifampicin–voriconazole combination should be managed as contraindicated or strongly avoided (VFEND, 2026). If rifampicin has recently been stopped, voriconazole should not be restarted immediately at the conventional dose. An approximately 7-day interval may be used as an initial observation-based reference, but it does not ensure complete de-induction; re-initiation should be guided by serial trough TDM, clinical response, toxicity, liver and renal function, inflammatory status, and the full concomitant-medication profile. The interval may need to be longer in individual patients, and empirical dose escalation should be avoided.

#### 3.4.2 Rifapentine

Rifapentine acts in the same direction as rifampicin and can reduce voriconazole exposure (National Clinical Research Center for Infectious Diseases/Shenzhen Third People’s Hospital, 2025), but evidence on the magnitude and duration of this interaction remains limited. A cohort study (Na et al., 2021) included 49 tuberculosis patients receiving voriconazole TDM, of whom 18 were receiving rifamycin coadministration and 31 were not. The mean trough concentration in the combination group was 1.39 ± 2.00 mg/L, lower than 2.82 ± 1.54 mg/L in the non-combination group, and the target attainment rates were 22.22% and 83.87%, respectively. Subgroup analysis showed mean trough concentrations of 3.37 ± 2.72 mg/L in the rifampicin subgroup (4 patients) and 0.82 ± 1.27 mg/L in the rifapentine subgroup (14 patients). However, only four patients were included in the rifampicin subgroup, and some samples were collected 6–9 days after rifampicin withdrawal, when enzyme induction may already have been resolving and voriconazole concentrations rising; the few high values could therefore substantially influence the mean. Available evidence generally suggests that rifampicin reduces voriconazole exposure more strongly than rifapentine, although direct head-to-head clinical comparisons are lacking. Rifapentine can nevertheless cause clinically significant voriconazole underexposure, and its induction effect may persist for several days or longer after withdrawal (Lu et al., 2024).

A cohort study (Lu et al., 2024) included 109 tuberculosis patients with chronic pulmonary aspergillosis and obtained 124 voriconazole concentrations; the study comprised four subgroups: 22 patients on rifampicin, 32 on rifapentine, 32 after rifampicin withdrawal, and 23 after rifapentine withdrawal. All 22 concentrations in the rifampicin group were below the therapeutic range, with a mean of (0.14 ± 0.26) mg/L; in the rifapentine group, only 6 of 32 concentrations were within the 1.0–5.5 mg/L therapeutic range, with a mean of (0.63 ± 0.97) mg/L. In two additional patients, the voriconazole concentrations before rifapentine were 1.38 and 2.52 mg/L, and after 7–10 days of rifapentine they fell to 0.25 and 0.72 mg/L, a reduction of approximately 71.4%–81.9%. These data support the view that rifapentine reduces voriconazole exposure less strongly than rifampicin but still to a clinically significant degree.

Case reports supplement the time course of the rifapentine interaction. A report (Ye et al., 2020) described a 69-year-old man with tuberculosis and fungal infection in whom the voriconazole trough concentration fell from 1.95 to 0.37 mg/L after 5 days of rifapentine; after rifapentine withdrawal and voriconazole dose adjustment, the concentration recovered to 2.18 mg/L. Another report (Ling et al., 2023) described an 85-year-old patient in whom the voriconazole trough concentration was 0.55 mg/L on day 3 after rifapentine withdrawal, below 0.5 mg/L on day 5, and reached 1.81 mg/L only on day 10. A recent case (Chen et al., 2026) showed a voriconazole trough concentration of 0.4 mg/L during rifapentine coadministration; after rifapentine withdrawal and an increase of voriconazole to 300 mg every 12 h, the concentration remained low in the short term but then rose to 3.0 mg/L on day 14 and 10.8 mg/L on day 18, exceeding the therapeutic window.

#### 3.4.3 Rifabutin

Rifabutin has a complex, bidirectional interaction with voriconazole (Li et al., 2017; i3 Pharmaceuticals, LLC, 2026). Rifabutin can induce voriconazole metabolism and lower voriconazole exposure, whereas voriconazole inhibits CYP3A4 and may increase exposure to rifabutin and 25-O-desacetyl-rifabutin (Blaschke and Skinner, 1996; Burman et al., 2001). Current U.S. voriconazole labeling lists coadministration with rifabutin as contraindicated. Rare off-label use described in case reports does not establish rifabutin as a safe alternative; management should follow the applicable local product label and involve specialist review.

A case report (Schwiesow et al., 2008) described a 30-year-old woman with mixed *Mycobacterium xenopi* and *Aspergillus* fumigatus infection receiving voriconazole and rifabutin among multiple drugs. During oral voriconazole treatment at 300 mg twice daily, trough concentrations were 0.22 and 0.12 mg/L, both below the report’s historical trough target of >0.5 mg/L. After voriconazole was increased to 300 mg orally three times daily, the trough concentration increased to 0.68 mg/L, exceeding that historical target. The observed rifabutin concentration in this single patient cannot quantify the reverse interaction or establish safety. The case supports concern for voriconazole underexposure and the need to monitor both drug exposure and toxicity after inadvertent or off-label coadministration.

A report by Debraine described voriconazole concentrations rising within a few days after rifampicin was replaced by rifabutin, as summarized in a review (Li et al., 2017). This secondary report does not establish rifabutin as safe. Healthy-volunteer data and limited clinical reports indicate that rifabutin can still reduce voriconazole exposure, while the reverse interaction may increase rifabutin or metabolite exposure. In view of current labeling, these reports should be treated as off-label or inadvertent-exposure evidence rather than support for routine substitution.

A retrospective study (Kim et al., 2025) included six patients receiving voriconazole and rifabutin; four experienced adverse reactions. Although voriconazole doses were approximately 1.81 times the recommended dose, concentrations initially remained below 1.0–5.5 mg/L. During septic shock, concentrations increased 13.7- to 36-fold relative to baseline in this small cohort. These observations cannot establish a predictable magnitude or isolate septic shock as the cause. Severe illness and inflammation may modify CYP-mediated metabolism and confound interpretation of the rifabutin effect; this is a potential modifier, not a definitive explanation for every individual case. Serial TDM, inflammatory markers, and clinical assessment are therefore preferable to fixed dose changes.

Current U.S. voriconazole labeling lists rifabutin coadministration as contraindicated. If inadvertent or unavoidable off-label exposure has already occurred, obtain urgent specialist review, follow local labeling, and monitor voriconazole trough concentrations serially together with liver function, QT interval, neuropsychiatric and visual symptoms, and rifabutin-related uveitis or myelosuppression; measure rifabutin or metabolite concentrations when feasible. Fixed empirical dose escalation should be avoided because the balance between induction, inflammation, and organ function can change rapidly.

### 3.5 Evidence on other antituberculosis drugs

#### 3.5.1 Ethambutol

The main clinical concern with the ethambutol–voriconazole combination is possible additive optic toxicity (Saxena et al., 2021). One case report (Orssaud et al., 2021) described a 15-year-old patient with cystic fibrosis and lung transplantation who developed visual dysfunction after ethambutol was added to voriconazole. Vision continued to deteriorate until voriconazole was also discontinued, after which visual function gradually recovered. The report and pharmacovigilance information raise a mechanistically plausible hypothesis that ethambutol may contribute to neuro-ocular toxicity, potentially through CYP2C19-related effects, but no voriconazole concentration or genotype was available. This single case cannot establish causality or a reproducible pharmacokinetic interaction; clinical management should emphasize ophthalmic assessment and individualized TDM when exposure variability is also suspected.

#### 3.5.2 Linezolid

Evidence for a linezolid–voriconazole interaction is limited and the mechanism is not established (Huo et al., 2024). A conference abstract (Idoate et al., 2019) described five patients in whom voriconazole clearance increased and four developed subtherapeutic concentrations during coadministration. These observations are suggestive of a potential exposure change, but the small, uncontrolled sample and conference-only reporting prevent causal attribution. An analysis of the U.S. Food and Drug Administration Adverse Event Reporting System (FAERS) (Shu et al., 2024) identified safety signals for the combination, but spontaneous reports cannot determine causality or the direction of concentration changes. Linezolid should therefore not be classified as a confirmed pharmacokinetic (PK) interaction comparable to rifampicin; if coadministration is unavoidable, use TDM together with monitoring for myelosuppression, neurotoxicity, liver injury, and treatment response.

#### 3.5.3 Other antituberculosis drugs

Besides rifamycins, ethambutol, and linezolid, direct human pharmacokinetic studies of other antituberculosis drugs such as isoniazid, pyrazinamide, and bedaquiline combined with voriconazole are generally limited. This section therefore distinguishes three categories of evidence: mechanistic inference based on metabolic enzymes; pharmacodynamic or toxicity-additive risks such as QT prolongation, hepatotoxicity, and nephrotoxicity; and potential pharmacokinetic interactions supported by individual case reports or *in vitro* studies.

Isoniazid inhibits CYP2C19 and CYP3A activity *in vitro* (Desta et al., 2001), so an increase in voriconazole exposure is mechanistically plausible. However, no direct human study has reported voriconazole trough concentrations, AUC, clearance, or dose-adjustment outcomes after the combination. Isoniazid should therefore be considered hypothesis-generating rather than a confirmed strong-interaction drug. TDM and liver-function monitoring are reasonable when patients have a CYP2C19 poor-metabolizer phenotype, hepatic dysfunction, severe illness, or polypharmacy, but routine empirical dose changes are not supported.

Pyrazinamide is mainly metabolized by hepatic amidase to pyrazinoic acid and further by xanthine oxidase-related pathways (Hussain et al., 2021). The main risk of combining pyrazinamide with voriconazole is not a clear CYP-mediated pharmacokinetic interaction but additive hepatotoxicity. Direct clinical evidence that pyrazinamide changes voriconazole concentrations is lacking, so clinical management should emphasize serial liver-function monitoring, symptom recognition, and voriconazole TDM when needed to assess exposure.

Bedaquiline is mainly metabolized by CYP3A4 (van Heeswijk et al., 2014); voriconazole could theoretically increase bedaquiline exposure through CYP3A4 inhibition. No direct human pharmacokinetic study of the combination is available, so this remains a mechanistically plausible concern rather than an established interaction. Empirical voriconazole dose adjustment is not recommended. Attention should focus on bedaquiline-related toxicity, liver-function abnormalities, QT prolongation, and serial electrocardiography (ECG) and electrolyte assessment. Delamanid, clofazimine, moxifloxacin, and levofloxacin likewise lack clear direct PK evidence with voriconazole; the principal concern is additive QT prolongation and related pharmacodynamic risk (Abdelwahab et al., 2021; Jin et al., 2023).

Pretomanid metabolism partially involves the CYP3A pathway. Existing studies show that CYP3A inducers reduce pretomanid exposure, whereas clinical evidence that CYP3A inhibitors increase its exposure is relatively limited; pretomanid alone has not shown a clinically concerning QT-prolonging effect, and when combined with moxifloxacin, the QT changes are mainly consistent with the effect of moxifloxacin (Li et al., 2021). The pretomanid–voriconazole combination should therefore still be regarded as lacking direct interaction data and requiring close monitoring. If the regimen also contains bedaquiline, clofazimine, or fluoroquinolones, the QT interval and hepatotoxicity should be the priority.

Cycloserine, terizidone, ethionamide, protionamide, para-aminosalicylic acid, amikacin, and streptomycin currently lack direct human PK/TDM studies with voriconazole. For cycloserine and terizidone, central nervous system adverse reactions deserve more attention; ethionamide, protionamide, and para-aminosalicylic acid require attention to gastrointestinal reactions, liver function abnormalities, and thyroid dysfunction; amikacin and streptomycin are mainly eliminated by the kidneys, and the management focus is nephrotoxicity, ototoxicity, and electrolyte abnormalities rather than empirical adjustment of the voriconazole dose.

Among carbapenems, meropenem is a notable but uncertain signal. A case report (Mahmoudi et al., 2017) described markedly reduced voriconazole clearance in a patient with renal failure receiving high-dose meropenem, and subsequent *in vitro* work suggested CYP2C19/CYP3A4 inhibition. This evidence is hypothesis-generating and cannot establish that meropenem reproducibly changes voriconazole exposure. In critically ill patients, patients with renal dysfunction, or patients receiving high-dose meropenem, TDM and toxicity monitoring are reasonable; routine empirical dose reduction is not justified. No comparable direct evidence is available for imipenem/cilastatin.

In summary, most non-rifamycin antituberculosis drugs lack direct human PK/TDM evidence with voriconazole. Ethambutol, linezolid, isoniazid, bedaquiline, and meropenem should be described as potential, mechanistically plausible, or hypothesis-generating interactions or toxicity signals, not established causal relationships. Management should use risk-matched monitoring: serial voriconazole TDM when exposure variability is clinically relevant; liver tests for additive hepatotoxicity; ECG and electrolytes for QT-prolonging combinations; and ophthalmic, neurologic, hematologic, renal, or auditory assessment according to the drugs used.

The characteristics of the 17 primary clinical studies are summarized in Table 3. Table 4 presents selected clinical reports together with supplementary pharmacovigilance evidence that was used for signal detection rather than counted among the 17 primary studies. Overall, the evidence base remains limited and is composed mainly of case reports, small case series, retrospective TDM studies, and observational cohorts. Rifamycins provide the clearest and most clinically important evidence, whereas findings for ethambutol, linezolid, and other non-rifamycin agents should be interpreted as limited, mechanistic, or signal-generating evidence.

**TABLE 3:** Basic characteristics of the 17 primary clinical studies.

Author, year	Anti-TB drug(s)	Design	Sample size	Age, years	Sex, M/F	Weight/BMI	VORI route	Intervention	Control
Geist et al., 2007	Rifampicin	Case report	1	NR	NR	NR	Oral	VORI + RIF	VORI alone (baseline)
Cojutti et al., 2016	Rifampicin	Retrospective cohort	2	NR	NR	NR	Intravenous and oral	VORI + RIF	NR
Farrokh and Avdic, 2019	Rifampicin	Case report	1	32	1/0	72/NR	Intravenous	VORI after RIF withdrawal	VORI alone (baseline)
Lu et al., 2019	Rifampicin	Single-arm study	18	58.1 ± 12.5	16/2	NR	Intravenous	VORI + RIF	VORI alone (historical)
Wang et al., 2019	Rifampicin	Case report	1	40	1/0	50/17.3	Intravenous	VORI + RIF	VORI alone (baseline)
Lin et al., 2020	Rifampicin	Case report	1	56	1/0	50/19.5	Intravenous and oral	VORI after RIF withdrawal	VORI alone (baseline)
Lin et al., 2021	Rifampicin	Case series	2	NR	NR	NR	Intravenous	VORI + RIF	VORI alone
Chen et al., 2023	Rifampicin	Retrospective cohort	15	51.5 ± 16.0	14/1	47.9 ± 4.2/NR	Intravenous and oral	VORI + RIF	VORI alone
Sternberg et al., 2025	Rifampicin	Case report	1	36	1/0	NR	Intravenous and oral	VORI + RIF (then VORI switched to isavuconazole/posaconazole + RIF to RFB)	VORI alone (baseline)
Na et al., 2021	Rifampicin	Cohort study	4	(Overall cohort; subgroup-specific value not reported)	(Overall cohort; subgroup-specific value not reported)	(Overall cohort; subgroup-specific value not reported)/(overall cohort; subgroup-specific value not reported)	Intravenous and oral	VORI + RIF	VORI alone
Na et al., 2021	Rifapentine	Cohort study	14	(Overall cohort; subgroup-specific value not reported)	(Overall cohort; subgroup-specific value not reported)	(Overall cohort; subgroup-specific value not reported)/(overall cohort; subgroup-specific value not reported)	Intravenous and oral	VORI + RPT	VORI alone
Ye et al., 2020	Rifapentine	Case report	1	69	1/0	71/24.6	Intravenous and oral	VORI + RPT	VORI alone (baseline)
Ling et al., 2023	Rifapentine	Case report	1	85	1/0	35/12.9	Intravenous	VORI after RPT withdrawal	VORI alone (baseline)
Lu et al., 2024	Rifapentine	Retrospective cohort	32	64.0 ± 17.0	25/7	NR	Intravenous and oral	VORI + RPT	VORI alone (historical)
Chen et al., 2026	Rifapentine	Case report	1	53	1/0	45/NR	Intravenous	VORI + RPT (then RPT stopped)	VORI alone (baseline)
Schwiesow et al., 2008	Rifabutin	Case report	1	30	0/1	54.5/NR	Intravenous, then oral	VORI + RFB	No comparator
Kim et al., 2025	Rifabutin	Retrospective cohort	6	NR	4/2	NR	Intravenous and oral	VORI + RFB	VORI alone (historical)
Orssaud et al., 2021	Ethambutol	Case report	1	15	1/0	NR	NR	VORI + EMB	VORI alone

NR, not reported; TB, tuberculosis; M = male; F = female; VORI, voriconazole; RIF, rifampicin; RPT, rifapentine; RFB, rifabutin; EMB, ethambutol; LZD, linezolid; BMI, body mass index. Weight is reported in kg and BMI, in kg/m^2^. Table 3 demographic values are overall-cohort values unless a subgroup-specific value is explicitly reported.

**TABLE 4:** Selected clinical reports and supplementary pharmacovigilance evidence on antituberculosis drug interactions with voriconazole.

Author, year	Drugs	Dosing and sequence	Outcomes	Comments
Geist et al., 2007	RIF + VORI	VORI: 400 mg loading dose then 200 mg twice daily orally; RIF 600 mg once daily added on day 7; coadministered for 30 days	VORI exposure decreased by 99%; Cmax: 3.92 to 0.038 mg/L; AUC: 27.4 to 0.145 h·mg/L; metabolite/parent ratios increased 45- to 422-fold	Strong induction; exposure failure; avoid coadministration
Farrokh and Avdic, 2019	RIF (discontinued) to VORI	RIF given empirically then discontinued; VORI started at 8.5 mg/kg intravenously every 12 h due to residual RIF induction; doses adjusted: 5.5 to 8.5 to 6.5 q8h to 8.5 q8h	VORI trough concentrations: Day 4 was 2.0, day 24 was below 0.1, day 48 was 1.1, day 74 was 27.2 (supratherapeutic, saturable metabolism); CSF levels were approximately 50% of serum (3.9–4.2 mg/L)	Residual induction and nonlinear recovery; serial TDM required
Wang et al., 2019	VORI to RIF + EMB + LZD + PZA to RIF stopped	VORI 200 mg every 12 h intravenously alone (Day 7 trough 5.98 mg/L); then RIF 0.45 g once daily plus EMB, LZD, PZA added; RIF discontinued after 3 days	With RIF on day 3: VORI trough was 0.75 mg/L (decreased by 87.5%); after RIF stop: Day 3 was 0.90, day 10 was 2.78 mg/L	Rapid exposure decrease after RIF; recovery remained delayed after withdrawal
Lin et al., 2021	RIF + INH + PZA + EMB + VORI	RIF 0.3 g twice daily intravenously plus INH, PZA, EMB; VORI 200 mg every 12 h intravenously added	VORI trough on day 4: 0.2 mg/L; RIF discontinued; day 7 after discontinuation: 1.0 mg/L	Low trough during RIF and recovery after withdrawal; TDM needed
Sternberg et al., 2025	RIF to RFB + VORI to isavuconazole to posaconazole + INH + levofloxacin + LZD	Initial: RIF 600 mg intravenously + INH 1200 mg + PZA + EMB + levofloxacin + LZD; switched to VORI with RFB (undetectable); then isavuconazole; finally posaconazole 300 mg twice daily + RFB 450 mg + INH + levofloxacin	VORI was undetectable with RFB; posaconazole achieved therapeutic levels (>2 mg/L) at 300 mg twice daily; clinical improvement and discharge	Single-case evidence; RIF-to-RFB substitution did not restore VORI exposure, and an alternative triazole with TDM was required
Ye et al., 2020	RPT + VORI	VORI 200 mg every 12 h intravenously (no loading dose); RPT 600 mg twice weekly added; VORI increased to 0.3 g every 12 h, then 0.3 g every 8 h; RPT discontinued; VORI reduced	VORI alone: trough 1.95 mg/L; with RPT on day 5: 0.37 mg/L; after RPT stop: 2.18 mg/L; later increased to 6.85 mg/L after dose reduction back to 0.2 g every 12 h	RPT markedly reduced VORI exposure; delayed de-induction after withdrawal can cause overexposure if an escalated dose is maintained
Ling et al., 2023	RPT (stopped) to VORI	RPT 450 mg twice weekly discontinued; VORI 150 mg every 12 h started (later increased to 200 mg every 8 h)	Day 3 after RPT stop: VORI trough 0.55 mg/L; day 5: below 0.5 mg/L; day 10: 1.81 mg/L at 200 mg every 8 h; AST elevated during treatment	Residual RPT induction may persist after withdrawal; use serial TDM and liver-function monitoring
Chen et al., 2026	RPT + VORI	Patient on RPT 0.45 g twice weekly; VORI 300 mg loading then 200 mg every 12 h; RPT discontinued on day 7; VORI increased to 300 mg every 12 h; later held then restarted at 200 mg every 12 h	With RPT: VORI trough 0.4 mg/L on day 7; after RPT stop and VORI increase: Day 10 was 0.4, day 14 was 3.0, day 18 was 10.8 (toxic); re-initiation: Day 10 was 0.1, day 14 was 4.3 mg/L	Delayed de-induction after RPT withdrawal; maintaining an escalated VORI dose can cause marked overexposure and toxicity
Schwiesow et al., 2008	RFB + VORI	VORI 300 mg intravenously every 12 h at transfer, subsequently 300 mg orally twice daily during TDM; RFB 150 mg orally once daily. Because of low VORI trough concentrations, VORI was increased to 300 mg orally three times daily; also EMB, azithromycin, and moxifloxacin	During oral VORI 300 mg twice daily, trough concentrations were 0.22 mg/L on day 7 and 0.12 mg/L on day 21. After escalation to 300 mg orally three times daily, the trough concentration was 0.68 mg/L on day 38, above the report’s historical trough target of >0.5 mg/L. RFB post-dose concentrations were 0.32 mg/L at both 2–3 h and 6–7 h on day 21 (target 0.3–0.9 mg/L); ethambutol and moxifloxacin concentrations were below their target ranges initially	Bidirectional RFB–VORI interaction; current labeling contraindicates coadministration, and both exposures and toxicity require review after inadvertent use
Orssaud et al., 2021	EMB + VORI	VORI plus EMB 22.2 mg/kg/day; EMB discontinued initially, VORI continued; VORI later discontinued	Toxic optic neuropathy developed; visual acuity dropped to 20/145–20/200; recovered to 20/20 over 1 year after VORI discontinuation	Possible additive optic toxicity; causality and a pharmacokinetic interaction were not established
Cojutti et al., 2016	RIF + VORI (also other inducers)	VORI doses normalized to 4 mg/kg every 12 h; cotreatments included RIF (2 patients), methylprednisolone, dexamethasone, etc.	RIF decreased dose-normalized VORI trough by average 3.18 mg/L; CYP inhibitors (proton pump inhibitors) increased risk of trough above 5.5 mg/L by 23-fold (OR 23.22); PPI plus inducers showed no significant increase	RIF and other CYP-active comedications altered VORI exposure; confounding limits attribution to a single drug
Lu et al., 2019	RIF + VORI; VORI after RIF stop	Various VORI regimens (mainly 200 mg every 12 h intravenously) with RIF (mostly 450–600 mg once daily) or after RIF discontinuation	RIF + VORI (n = 18): 94.44% had trough below 1.0 mg/L, 72.22% below 0.16 mg/L; after RIF stop (n = 19): within 6 days, 91.67% below 1.0; at 7 days or more, 83.33% above 1.0 mg/L	RIF caused marked underexposure and recovery after withdrawal was variable; serial TDM is required
Chen et al., 2023	RIF + VORI	VORI 200 mg every 12 h (loading 300–400 mg) with RIF 0.45–0.6 g once daily; some without RIF; some critically ill with SOFA score greater than 10	RIF group: mean trough 1.58 mg/L, attainment rate 26.7%, 66.7% below 1.0 mg/L. Non-RIF: mean 3.67 mg/L, attainment 82.4%. All 10 subtherapeutic cases sampled within 7 days of RIF stop. Three critically ill patients (SOFA >10) maintained trough >1.0 mg/L despite RIF	RIF-associated underexposure; critical illness may modify the magnitude and direction of observed concentrations
Na et al., 2021	RIF + VORI; RPT + VORI	VORI 200 mg every 12 h (mainly) with RIF 0.45–0.6 g once daily or RPT 0.45–0.6 g twice weekly; some VORI alone	RIF group: mean trough 3.37 mg/L, attainment 25.0%. RPT group: mean 0.82 mg/L, attainment 21.4%. Non-rifamycin: mean 2.82 mg/L, attainment 83.87%. Six ADRs (all non-rifamycin) with mean trough 5.96 mg/L	Both RIF and RPT were associated with low target attainment; the RIF subgroup was small (n = 4) and demographic data were cohort-level
Lu et al., 2024	RIF + VORI; RPT + VORI; VORI after discontinuation	VORI 200 mg every 12 h; RIF 450–600 mg once daily or RPT 450–600 mg twice weekly; TDM measured during and after discontinuation	RIF + VORI: all 22 concentrations were below the therapeutic range; mean 0.14 ± 0.26 mg/L. RPT + VORI: mean 0.63 ± 0.97 mg/L; RPT reduced VORI by more than 70% in 2 patients. After RIF stop: 7 days or more recovery (66.67% above 1.0 mg/L). After RPT stop: 6 days or more recovery (100% within therapeutic range)	RIF/RPT caused marked underexposure and different recovery timelines; serial TDM is needed during and after exposure
Kim et al., 2025	RFB + VORI	VORI doses titrated (average 724 mg/day, 1.81-fold higher than standard) with RFB 300 mg once daily; TDM and adverse drug reaction monitoring	4 of 6 patients had adverse drug reactions: neurological (hallucinations, delirium), QTc prolongation, elevated AST/ALT, severe nausea; VORI remained subtherapeutic (0.1–0.6 mg/L) despite higher dose; during septic shock, VORI increased 13.7- to 36-fold (up to 10.6 mg/L)	RFB-associated underexposure and adverse reactions; abrupt VORI overexposure was observed during septic shock in this small cohort; causality is uncertain
Shu et al., 2024	LZD + VORI (FAERS analysis)	FAERS database analysis; LZD combined with VORI or other antibiotics; no specific dosing details	LZD + VORI: 1,726 adverse event reports; positive safety signals for condition aggravation, drug resistance, QTc prolongation (all 4 statistical models); multiple organ dysfunction and septic shock strongly correlated; sex and age influenced adverse events	Pharmacovigilance signal only; causality and the direction of any pharmacokinetic interaction cannot be established

VORI, voriconazole; RIF, rifampicin; RPT, rifapentine; RFB, rifabutin; EMB, ethambutol; INH, isoniazid; LZD, linezolid; PZA, pyrazinamide; TDM, therapeutic drug monitoring; Cmin = minimum (usually trough) concentration; Cmax = peak concentration; AUC, area under the concentration–time curve; CL/F = apparent oral clearance; CSF, cerebrospinal fluid; AST, aspartate aminotransferase; ALT, alanine aminotransferase; FAERS, U.S. Food and Drug Administration Adverse Event Reporting System; QTc, corrected QT interval. SOFA, sequential organ failure assessment; ADR, adverse drug reaction; OR, odds ratio; PPI, proton pump inhibitor; CYP, cytochrome P450; NR, not reported.

Therapeutic drug monitoring should be matched to the clinical question and interpreted with reference to antifungal TDM guidance (Ashbee et al., 2014). It is strongly recommended when clinically important rifamycin exposure is suspected, including inadvertent rifampicin or rifabutin exposure and rifapentine coadministration; after discontinuation of rifampicin or rifapentine when voriconazole is being restarted; and after major voriconazole dose changes or unexpected clinical response. TDM is reasonable when meropenem, isoniazid, severe illness, organ dysfunction, or extensive polypharmacy could materially affect exposure. Because voriconazole has high interindividual variability, TDM is useful in most high-risk patients, especially when efficacy is inadequate or toxicity is suspected. Table 5 distinguishes pharmacokinetic interactions from pharmacodynamic or additive toxicity risks. Voriconazole TDM does not replace toxicity monitoring: ECG and electrolytes are needed for QT risk, liver tests for hepatotoxicity, CBC for marrow toxicity, and ophthalmic or neurologic assessment for visual or neuropsychiatric effects.

**TABLE 5:** Distinguishing pharmacokinetic interactions from pharmacodynamic or additive toxicity risks.

Interaction type	Examples in this review	What can be inferred	Clinical management
Pharmacokinetic interaction	RIF/RPT/RFB effects on VORI exposure; possible meropenem or INH signals	Changes in trough, AUC, Cmax, clearance, or metabolite exposure when measured; strength depends on direct human PK data	Avoid RIF–VORI and RFB–VORI coadministration in accordance with current labeling; avoid RPT where feasible; use serial TDM after recent or inadvertent rifamycin exposure; do not use routine empirical adjustment for unconfirmed signals
Pharmacodynamic or additive toxicity	QT prolongation with VORI plus bedaquiline, delamanid, clofazimine, or fluoroquinolones; optic toxicity with EMB	Additive risk can occur without a change in VORI concentration	ECG, electrolytes, ophthalmic assessment, liver tests, CBC, and symptom monitoring according to the agents used
Shared toxicity or confounding	Hepatotoxicity, neurotoxicity, critical illness, inflammation, renal dysfunction, polypharmacy	Observed concentrations or adverse events may reflect multiple causes; causality is uncertain	Interpret TDM with organ function, inflammatory status, adherence, disease severity, and concomitant medicines
Mechanistic or hypothesis-generating evidence	*In vitro* CYP inhibition/induction; isolated case reports; pharmacovigilance signals	Supports a biologically plausible hypothesis but does not establish a clinical interaction	Avoid unvalidated dose changes; intensify monitoring when the clinical consequence would be serious

Abbreviations: RIF, rifampicin; RPT, rifapentine; RFB, rifabutin; VORI, voriconazole; INH, isoniazid; EMB, ethambutol; AUC, area under the concentration-time curve; Cmax = maximum concentration; PK, pharmacokinetic; TDM, therapeutic drug monitoring; ECG, electrocardiography; CBC, complete blood count; CYP, cytochrome P450.

## 4 Discussion

As summarized in Tables 3, 4, the most robust evidence concerns the rifamycins, particularly rifampicin, which consistently produces a marked decrease in voriconazole concentrations across study designs. Rifapentine and rifabutin may also reduce voriconazole exposure, although the magnitude and recovery time vary and rifabutin has a clinically important bidirectional risk. In contrast, evidence for non-rifamycin antituberculosis drugs is sparse and heterogeneous; most findings are suggestive, mechanistically plausible, or hypothesis-generating and should not be interpreted as definitive pharmacokinetic interactions.

Voriconazole is an important treatment for severe fungal infections such as invasive aspergillosis, but its CYP2C19, CYP2C9, and CYP3A4 metabolism produces wide interindividual variability and a relatively narrow therapeutic window (Hyland et al., 2003; Dolton et al., 2012; Moriyama et al., 2017). The strongest and most clinically informative evidence concerns rifamycins. For ethambutol, linezolid, and other non-rifamycin drugs, available observations should be interpreted as potential safety or exposure signals rather than proof of a causal interaction.

Rifampicin is the antituberculosis drug with the most clearly defined and strongest effect on voriconazole exposure (AX Pharmaceutical Corp, 2024; VFEND, 2026). Case studies, retrospective TDM studies, and cohort data consistently show profound underexposure that conventional dose escalation cannot reliably offset. After withdrawal, concentrations often remain ineffective during the first 6 days and are more likely to recover at 7 days or later, but individual cases show persistence for 10 days or longer. Thus, approximately 7 days should be presented only as an observed minimum in some datasets; it is not a universal washout rule, and resolution should be assessed with serial TDM and patient-specific clinical factors.

Evidence on the rifapentine–voriconazole interaction is less extensive than that for rifampicin but equally clinically relevant (VFEND, 2026). Previous studies have shown that rifapentine markedly reduces voriconazole trough concentrations, with reductions exceeding 70% in some patients; after rifapentine withdrawal, recovery of voriconazole concentrations is also delayed. Because rifapentine has a long half-life, is often given intermittently, and patient age, liver function, inflammatory state, and concomitant medication can all affect voriconazole exposure, empirical dose escalation is not reliable. Existing cases (Lu et al., 2024; Chen et al., 2026) show that if a high voriconazole dose is maintained after induction has resolved, concentrations can rapidly rise above the upper limit of the therapeutic window, increasing the risk of hepatotoxicity, neurotoxicity, and visual abnormalities.

Rifabutin is not a demonstrably safe alternative to rifampicin. It may lower voriconazole exposure through induction, while voriconazole may increase rifabutin and metabolite exposure, with risks including uveitis, myelosuppression, and liver abnormalities (i3 Pharmaceuticals, LLC, 2026). Current U.S. voriconazole labeling lists coadministration as contraindicated. Case reports and small observational studies should be interpreted as off-label experience, not evidence of routine substitutability; inadvertent exposure warrants specialist assessment, serial TDM, and toxicity monitoring in accordance with local labeling.

Evidence for interactions between non-rifamycin antituberculosis drugs and voriconazole is insufficient for most agents. Ethambutol case reports and *in vitro* studies suggest possible additive visual toxicity, but do not prove a stable increase in voriconazole exposure. Linezolid observations and pharmacovigilance signals suggest possible exposure changes or multi-system adverse events, but confounding by disease severity and polypharmacy cannot be excluded. Isoniazid, bedaquiline, and meropenem have mechanistic or isolated clinical signals without confirmatory human PK data. Delamanid, clofazimine, and fluoroquinolones primarily raise pharmacodynamic QT concerns, whereas pyrazinamide and other agents require toxicity-focused monitoring. These findings are hypothesis-generating and should not drive unvalidated empirical voriconazole dose changes.

For non-rifamycin agents, management should therefore emphasize agent-specific toxicity surveillance and clinically indicated TDM rather than unvalidated empirical voriconazole dose changes.

Management should be centered on risk stratification. Voriconazole coadministration with rifampicin or rifabutin should be avoided in accordance with current labeling; rifapentine also causes clinically important underexposure and should be avoided where feasible. After rifampicin or rifapentine discontinuation, an approximately 7-day interval is only an observation-based starting reference, not a guaranteed washout period. Serial trough TDM should guide re-initiation and subsequent dose changes, and guideline-concordant alternative antifungal coverage should be maintained while clinically important induction persists. Decisions should incorporate infection site and severity, pathogen and susceptibility, liver and renal function, age, inflammatory state, CYP2C19 phenotype or genotype, concomitant drugs, and serial efficacy and safety data. When a rifamycin regimen cannot be changed, select an alternative antifungal according to current disease-specific guidance; echinocandin monotherapy should not be assumed to be an equivalent first-line substitute for invasive aspergillosis (Patterson et al., 2016). The management algorithm is shown in Figure 2.

**FIGURE 2:**
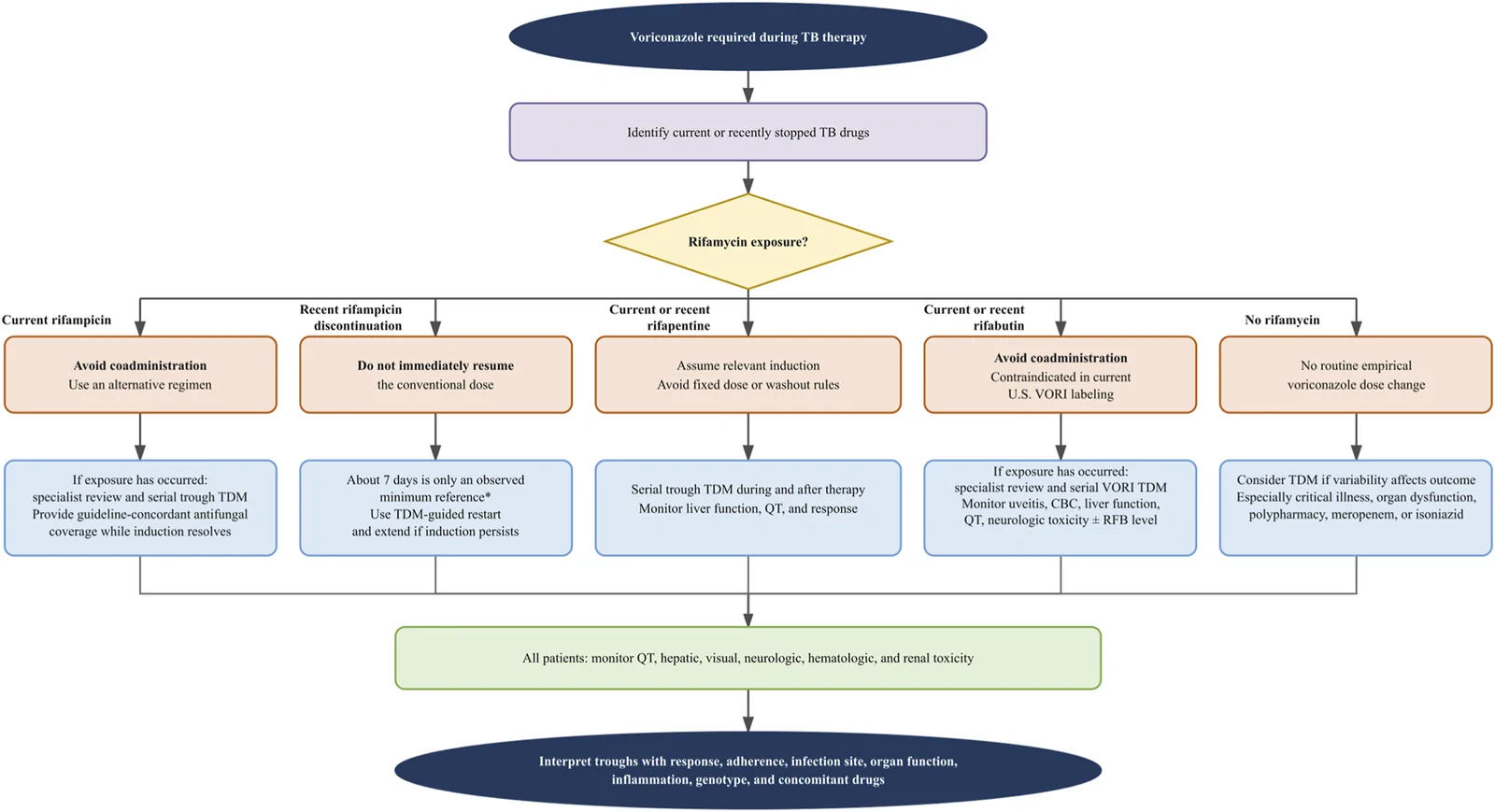
Clinical management algorithm for voriconazole with rifamycin and non-rifamycin antituberculosis drugs. Abbreviations: CBC, complete blood count; QT, QT interval; RFB, rifabutin; TB, tuberculosis; TDM, therapeutic drug monitoring; VORI, voriconazole. Approximately 7 days after rifampicin discontinuation is an observed minimum reference in some datasets, not a guaranteed washout period. Current U.S. voriconazole labeling lists coadministration with rifampicin and rifabutin as contraindicated; local labeling should be followed.

## 5 Limitations

This review has several limitations. First, the included studies were mainly case reports, case series, retrospective TDM studies, and small observational cohorts; differences in disease severity, dosing, sampling, and outcome definitions prevented quantitative pooling. Second, publication bias, selective reporting, and small-study effects are likely, but could not be formally assessed. Third, the absence of control groups, incomplete adjustment for confounding, and inconsistent reporting of genotype, inflammation, albumin, adherence, organ function, and concomitant medications limit causal inference. Pharmacovigilance data are useful for signal detection but cannot establish causality or direction of pharmacokinetic change. Finally, most studies focused on concentrations rather than fungal and tuberculosis outcomes or long-term safety. These limitations support cautious, hypothesis-generating interpretation and the need for prospective multicenter studies. Future studies should prospectively characterize drug-specific induction and de-induction time courses, prespecify sampling schedules and clinical endpoints, and evaluate exposure-guided management across *CYP2C19* genotypes and inflammatory states.

## 6 Conclusion

The strongest clinical evidence supports avoiding voriconazole with rifampicin and following current labeling for rifabutin, which lists coadministration as contraindicated in the United States. Rifapentine also causes clinically important and sometimes prolonged underexposure; if recent exposure cannot be avoided, serial TDM is required during dose adjustment and after withdrawal. For non-rifamycin antituberculosis drugs, evidence is generally mechanistic or signal-generating, so monitoring should target plausible exposure and toxicity risks rather than rely on unvalidated empirical voriconazole dose changes.
